# Evaluation of the Clinical Safety of the Low‐Cost Warburg Therapy for the Treatment of Patients With Advanced Cancers

**DOI:** 10.1002/cam4.70469

**Published:** 2024-12-04

**Authors:** Peihua Lu, Tom Tsang, Michael S. Badowski, Michael E. Pennington, Linda C. Meade‐Tollin

**Affiliations:** ^1^ Department of Hematology and Oncology The Affiliated Wuxi People's Hospital of Nanjing Medical University, Wuxi People's Hospital, Wuxi Medical Center, Nanjing Medical University Wuxi China; ^2^ School of Medicine Jiangnan University Wuxi China; ^3^ American Goodwill Mission to China Inc. 501(c) (3) Tucson Arizona USA; ^4^ Warburg Medical Chongqing China; ^5^ Department of Immunobiology University of Arizona Tucson Arizona USA; ^6^ Department of Surgery University of Arizona Tucson Arizona USA

## Abstract

**Background:**

Rising cancer care costs are becoming cost prohibitive for lower income people worldwide. We developed the Warburg protocol as a low‐cost option for the treatment of cancer that was inspired. It was developed to exploit an Achilles heel which is a hallmark of cancer cells; the metabolic requirement for higher levels of glucose than normal cells.

**Objective:**

The purpose of this report is to assess the clinical safety and affordability of the Warburg therapy as an option for patients with advanced cancers.

**Methods:**

Between 2021 and 2023, 251 patients with advanced cancers received a total of 8542 treatments with the Warburg therapy. To restrict the supply of blood glucose to cancerous tumors, regular human insulin was administered (IV) sufficient to reduce blood glucose concentrations to hypoglycemic levels for 40–60 min. Subroutine doses of fluorouracil and cyclophosphamide were administered intravenously during this hypoglycemic period. Food or intravenous glucose was given as needed to return blood glucose to euglycemic levels after treatment. Patient symptoms, status, vitals, blood glucose, and hypoglycemic symptoms were monitored throughout treatment. Various blood parameters were measured before and after patients' course of treatment.

**Results:**

There were no irreversible adverse reactions in advanced tumor patients of different ages and different cancer types after treatment. There was no significant fluctuation in blood glucose levels in diabetic and non‐diabetic patients after treatment, and the weight, vital index and blood biochemical index of patients before and after multiple treatments exhibited little variation.

**Conclusion:**

Warburg therapy for the treatment of advanced tumors is clinically feasible, and safe for multiple treatments. It is inexpensive and widely applicable to different patient groups.

## 1 Introduction

Recent global cancer statistics report that one‐sixth of global deaths are attributed to cancer, and most cancer deaths occur in low‐ and middle‐income countries [1]. A recent study by Chen et al. estimated the total cancer economic impact from 2020 to 2050 will be $25.2 trillion international dollars worldwide with China facing the highest economic burden at $6.1 trillion followed by the United States at $5.3 trillion and India at $1.4 trillion [2]. Cancer care costs in China can total more than USD 10,000 per year and often exceed half the patients' annual income [3]. In the United States, cancer care costs have increased 5–10‐fold in the past 20 years [4] and a 2023 study found the monthly average drug price at USD 24,444 with increasing costs having a weak correlation to improved overall survival [5]. These high cancer care costs have devastating financial effects on patients, with many depleting their life assets and going into bankruptcy [6]. There is a critical need for the development of affordable cancer care, especially for lower‐income patients and the global south.

In order to fulfill the need for affordable cancer care, we developed the “Warburg therapy,” an innovative treatment protocol. The Warburg therapy was inspired by Nobel Laureate Otto Warburg's 1927 report that the metabolism of cancer cells consumes far greater amounts of glucose than normal cells [7]. This metabolic shift, referred to as the “Warburg Effect,” is a hallmark of cancer [8]. In his report, Warburg found changing glucose and oxygen supply to tumors highly affects the metabolism of cancer cells and their survivability. Additionally, this high glucose metabolism is found to contribute to cancer cell resilience and drug resistance making this hallmark of cancer a major therapeutic target [9, 10, 11, 12, 13, 14, 15].

Continuing Otto Warburg's work on limiting glucose to starve cancer, we employ the use of insulin to induce short term hypoglycemia in the Warburg therapy protocol. The induced hypoglycemia limits the supply of blood glucose creating a hypoglycemic period during which chemotherapy drugs are administered. For the past decade, this therapy has been in use as care at multiple centers throughout China with previous reports on safety [16] and single incidence of efficacy in a case study report [17]. Under the guidance of the Chinese government's Poverty Alleviation Program, we report here an expanded clinical safety trial that assesses and establishes the Warburg therapy as a safe and affordable option for patients with advanced cancers.

## 2 Methods

The 251 participants in this study were recruited between 2021 and 2023 at the Shenqiu Xinglin Traditional Chinese Medicine Hospital under the approval of the Ethics Committee (SQ [2021‐6‐25]). Any patients with Stage III–IV cancers were included with only those with medication allergies excluded.

Patients were requested to only ingest water overnight to achieve consistent blood glucose levels. An intravenous push of insulin human regular was given to sufficiently lower blood glucose levels to a hypoglycemic state, typically below 2.8 mmol/L for non‐diabetics and 3.8 mmol/L for diabetics. On the onset of hypoglycemia, patients were administered bolus IV fluorouracil (250 mg/day) and cyclophosphamide (200 mg/day) combination therapy at subroutine doses. Thirty minutes post‐treatment, patients were given either IV glucose or food to raise blood glucose back to euglycemic levels. After admission, the patient was treated once a day for ten consecutive times as a course of treatment, and the next course of treatment was carried out after 2–3 months of rest. During treatment, the patient's blood glucose was measured every 10 min and vitals including pulse, ECG, temperature, and blood pressure were measured with a patient monitor. Clinical personnel monitored and recorded patients' symptoms of hypoglycemia and had IV glucose available to halt hypoglycemia if severe symptoms were present. Patient characteristics such as age, cancer type, changes in blood glucose, the total number of treatments, duration of individual treatments, the amount of insulin used, and diabetic status were recorded. Before the first course of treatment and after the end of the last course of treatment, the changes of blood routine and liver and kidney function were detected. Safety evaluation was performed based on vital signs, blood indices, body weight, and hypoglycemic symptoms.

## 3 Results

### 3.1 Patient Characteristics

Two hundred and fifty one patients with Stage III–IV cancers were enrolled with ages ranging from 11 to 95 years. Two‐thirds of them were above the age of 61. The study included both diabetic and non‐diabetic patients, with 80 patients, 31.9%, having type 2 diabetes mellitus. A total of 8542 treatments were performed over the patient population. Due to COVID‐19 restrictions, 92 patients underwent fewer than 10 treatment courses. Of the 159 patients that underwent more than 10 treatments, 117 (44.6%) patients received over 21 treatments and 22 patients underwent more than 81 treatments totaling 2713 treatments, the greatest number of treatments received per patient was 210. All advanced cancer types were eligible for the study with 25 different types, composing cancers of the digestive, urinary, reproductive systems and various others, the most common being lung, 24.3%, esophageal, 14.3%, and breast, 13.9% (Table 1).

**TABLE 1:** Patient demographics.

Characteristic	No. of patients	Percent
Age, years		
≤ 60	84	33.5
61–70	86	34.3
≥ 71	81	32.2
Diabetic state		
Non‐diabetic	171	68.1
Diabetes mellitus type 2	80	31.9
Number of treatments per patient		
≤ 10	92	36.7
11–20	42	16.7
21–40	57	22.7
41–80	38	15.1
≥ 81	22	8.8
Cancer type		
Lung cancer	61	24.3
Esophageal cancer	36	14.3
Breast cancer	35	13.9
Gastric cancer	21	8.4
Liver cancer	18	7.2
Colon cancer	19	7.6
Cervical cancer	7	2.8
Gallbladder	6	2.4
Lymphoma	6	2.4
Bone cancer	5	2.0
Renal cell carcinoma	5	2.0
Bladder cancer	4	1.6
Ovarian cancer	4	1.6
Pancreatic cancer	4	1.6
Endometrial carcinoma	4	1.6
Oral cancer	3	1.2
Prostate cancer	3	1.2
Thyroid carcinoma	2	0.8
Skin cancer	2	0.8
Nasal cancer	1	0.4
Laryngeal cancer	1	0.4
Eyelid cancer	1	0.4
Malignant tumor of the thoracic spine	1	0.4
Vaginal cancer	1	0.4
Bronchogenic carcinoma	1	0.4

### 3.2 Induced Hypoglycemia by Insulin in Chemotherapy Treatments

**FIGURE 1:**
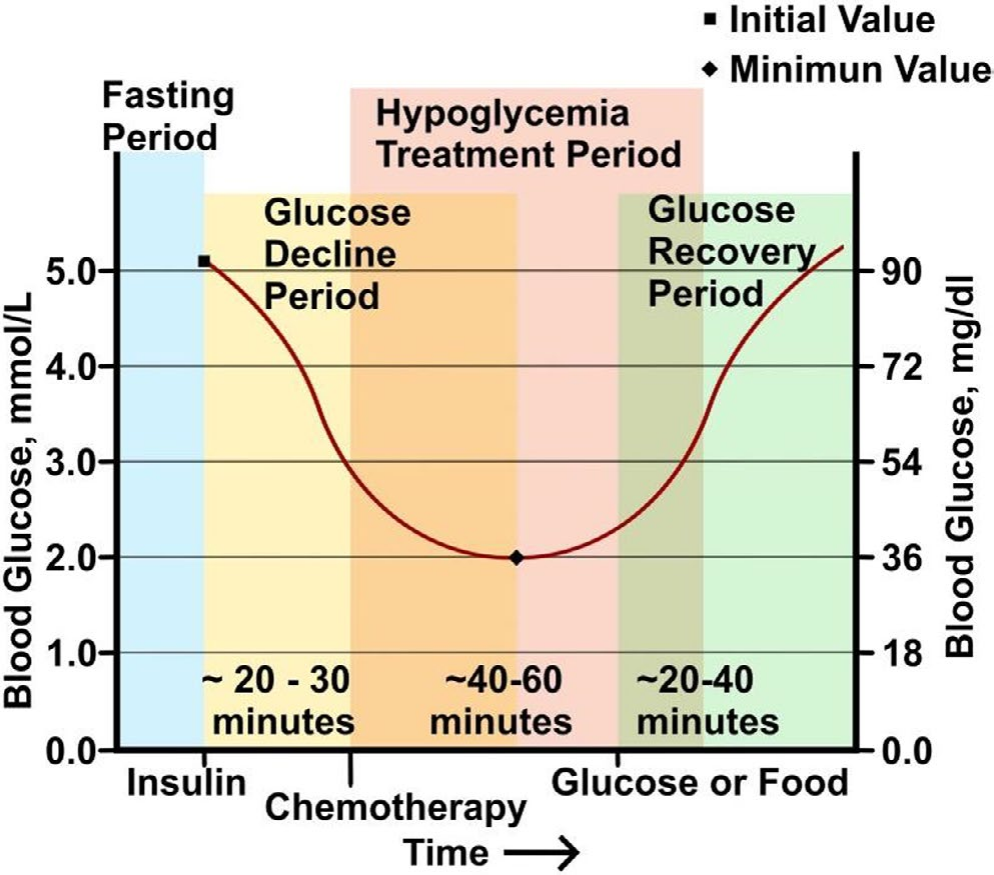
Diagram depicts the Warburg therapy. Fasting patients are given a dose of insulin sufficient to lower blood glucose levels in a typical case to approximately 2 mmol/L. After hypoglycemia is achieved, chemotherapy drugs are administered in a treatment period of typically 40–60 min. Blood glucose is returned to euglycemic levels with either IV glucose or food.

The Warburg Therapy uses insulin to induce hypoglycemia to create a low blood glucose window in which a combination of the chemotherapy drugs fluorouracil and cyclophosphamide are administered. The patient is generally kept in a hypoglycemic state for 40–60 min, after which IV glucose or food is given to raise blood glucose to euglycemic levels. See Figure 1 for a schematic depiction of the protocol.

The initial step of the Warburg Therapy is insulin delivery. Because each patient's initial blood glucose value and insulin sensitivity are different, the amount of insulin used is personalized and varies between patients. Typical insulin dosing was between 11 and 40 IU given in 6143 (71.9%) treatments. In 1338 (15.7%) treatments, the insulin dosage was less than 10 IU, and in 1061 (12.4%) treatments the insulin dosage was higher than 40 IU. In 19 (0.2%) treatments the insulin dosage was higher than 80 IU. The patient with the highest insulin dosage used 170 IU of insulin, but this patient's insulin use had a gradual reduction in subsequent treatments. None of the patients developed chronic insulin resistance defined as those requiring more than 200 IU of insulin per day.

The induction of hypoglycemia was monitored with blood glucose measurements in 10‐min intervals when fluorouracil and cyclophosphamide were administered to establish the period of hypoglycemia. In 68.1% of treatments, the duration of hypoglycemia in the target range is 41–60 min with only 2.5% lasting longer than 61 min. In 23.2% of treatments, hypoglycemia lasted for 21–40 min and only 6.1% of treatments lasted fewer than 20 min.

During the hypoglycemia period, the lowest blood glucose minima were in the range of 1.0–1.6 mmol/L in 22.4% of the treatments. Most treatments, 55.6%, fell into the range of 1.7–2.2 mmol/L with an additional 19.0% in the range of 2.3–2.8 mmol/L. In only 3.0% of treatments did blood glucose fail to fall below 2.9 mmol/L. This was in part due to the patients' fasting blood glucose value being greater than 7.0 mmol/L, which also affects the duration of the hypoglycemic period. At the end of chemotherapy, patients used food or IV glucose to restore euglycemia. In 98.6% of treatments, euglycemia was restored within 1 h. Only 1.4% of treatments required more than 1 h to restore euglycemia and none of the patients had any observed or reported after‐effects (Data S5, Table 2).

**TABLE 2:** Insulin dosage and induced hypoglycemia parameters.

Variable	Number of treatments	Percent
Insulin dose(UI)/treatment		
≤ 10	1338	15.7
11–20	3476	40.7
21–40	2667	31.2
41–80	1042	12.2
≥ 81	19	0.2
Duration of hypoglycemia during treatment		
Time (min)		
≤ 20	522	6.1
21–40	1985	23.2
41–60	5819	68.1
≥ 61	216	2.5
Distribution of blood glucose minima		
Blood glucose (mmol/L)		
1.0–1.6	1915	22.4
1.7–2.2	4749	55.6
2.3–2.8	1619	19.0
2.9–3.8	259	3.0
Time to return blood glucose to euglycemic levels		
Time (min)		
≤ 20	370	4.3
21–40	5334	62.4
41–60	2716	31.8
≥ 61	122	1.4

### 3.3 Safety Evaluation of Hypoglycemia

All the 8542 treatments were well tolerated with none of the patients experiencing serious irreversible phenomena such as shock, craniocerebral injury, deep coma or death due to hypoglycemia. No patients had acute or persisting sequelae requiring hospitalization from either the insulin‐induced hypoglycemia or fluorouracil and cyclophosphamide treatments nor were there any adverse events that necessitated admission of IV glucose to halt treatment. Weight was charted for the 159 patients with more than 10 treatments. One hundred and twenty nine patients had no significant fluctuation in body weight and 17 patients had a weight gain of more than 5%. Although 13 patients had a weight loss of more than 5%, only one person had a weight loss of more than 15% and no serious adverse morbidity was observed or reported (Data S6).

Throughout the treatment, clinical personnel were present to monitor blood glucose, record patients' symptoms in 10‐min intervals, and intervene with IV glucose if needed. Each instance of the various hypoglycemic symptoms was noted during each interval. There was no shortness of breath in all of the treatment sessions or changes in body temperature even in the patients experiencing perspiration. 3559 (41.7%) of the total 8542 treatments were symptom‐free. 4983 treatments showed some discomfort due to hypoglycemia with the following frequency of symptoms noted in the observation intervals: perspiration 12,716 (38.3%), palpitation 10,922 (32.9%), hypersomnia 7341 (22.1%), fatigue 1754 (5.3%), dizziness 439 (1.3%), and headache 21 (0.1%). In all cases symptoms were low grade without need for medical intervention. We found that the hypoglycemic symptoms had a temporal correlation when blood glucose dropped below 4 mmol/L and were generally confined to less than 60 min during the hypoglycemic period. Hypoglycemic symptoms persisted more than 60 min in only 48 of the 4983 symptomatic treatments (Table 3). Regardless of the duration of the symptoms, all symptoms returned to normal after food or IV glucose were administered at the end of treatment. There were no cases of worsening of hypoglycemic symptoms with the cumulative number of treatments.

**TABLE 3:** Frequency of hypoglycemic symptoms and duration during insulin induced hypoglycemia.

Symptom	Frequency recorded	Percent
Perspiration	12716	38.3
Palpitation	10922	32.9
Hypersomnia	7341	22.1
Fatigue	1754	5.3
Dizziness	439	1.3
Headache	21	0.1

Duration of symptoms	Number of treatments	Percent
Time (min)		
0	3562	41.7
≤ 20	1279	15.0
21–40	2751	32.2
41–60	902	10.6
≥ 61	48	0.6

Standard patient monitors recorded heart rate, systolic and diastolic blood pressure throughout the treatments and more than 75% of patients' vitals were stable during the treatment process. Some patients experienced changes of more than 15% in heart rate (11.2% decreased and 13.3% increased), and blood pressure, systolic (5.6% decreased and 2.8% increased) and diastolic (7.6% decrease and 3.6% increase), as the treatment progressed, but patients reported no special discomfort and did not require any intervention (Table 4). Their heart rate, systolic and diastolic blood pressures were restored to the pre‐treatment level when euglycemia was restored (Data S1–S4). There were no observable worsening trends in heart rate or blood pressure in patients who continued to receive multiple treatments.

**TABLE 4:** Heart rate and blood pressure fluctuation between the first and last recorded interval during a single day treatment.

	Heart rate		Systolic pressure		Diastolic pressure	
Type of variation	Number of patients	Percent	Number of patients	Percent	Number of patients	Percent
Decreased ≥ 15%	28	11.2	14	5.6	19	7.6
Variation = 0%–15%	188	75.5	228	91.6	211	84.7
Increased ≥ 15%	33	13.3	7	2.8	9	3.6

### 3.4 Assessment of Changes in Blood Parameters Post‐Therapy

Blood tests were performed in compliance with the patient's wishes and affordability. Initial screening indicated a minority of patients presented with abnormal blood parameters in the range of 7.2%–36.4% of the patient population per blood parameter either due to prior treatments or disease condition. On final screening, 71.8%–94.7% of the patient population per blood parameter was in normal ranges with some patients' blood parameters improving from abnormal to normal ranges, 3.8%–31.3%, with fewer than one in ten patients' blood parameters going from a normal to abnormal range, 1.9%–9.6%. 1.9%–18.6% of patients' blood parameters persisted in abnormal ranges throughout treatment. White blood cell (WBC) counts were in normal ranges in 90.2% of patients post‐treatment with WBC numbers improving in 70 of 82 patients which presented with abnormal WBC counts indicating the fluorouracil and cyclophosphamide chemotherapy treatments were well tolerated at the low doses used, and the immune system function was not severely compromised (Table 5).

**TABLE 5:** Comparison of normal and abnormal ranges in blood screening parameters from initial presentation to post final treatment in select patients.

	Screening, Initial		Screening, final		
Parameter	Normal	Abnormal	Normal	Abnormal	Reference value
WBC	143 (63.6%)	82 (36.4%)	203 (90.2%)	22 (9.8%)	4.00–10.00 (10^9^/L)
Hb	165 (73.3%)	60 (26.7%)	186 (82.7%)	39 (17.3%)	110–160 (g/L)
PLT	174 (77.3%)	51 (22.7%)	203 (90.2%)	22 (9.8%)	100–300 (10^9^/L)
ALT	132 (84.6%)	24 (15.4%)	135 (86.5%)	21 (13.5%)	0–40 (U/L)
AST	106 (67.9%)	50 (32.1%)	116 (74.4%)	40 (25.6%)	0–34 (U/L)
ALB	123 (78.8%)	33 (21.2%)	124 (79.5%)	32 (20.5%)	37–53 (g/L)
TBIL	110 (70.5%)	46 (29.5%)	112 (71.8%)	44 (28.2%)	5.1–19 (μmol/L)
DBIL	119 (76.3%)	37 (23.7%)	121 (77.6%)	35 (22.4%)	1.7–6.8 (μmol/L)
Urea	141 (91.0%)	14 (9.0%)	141 (91.0%)	14 (9.0%)	2.82–8.2 (mmol/L)
CR	130 (85.0%)	23 (15.0%)	132 (86.3%)	21 (13.7%)	45–104 (μmol/L)
UA	141 (92.8%)	11 (7.2%)	144 (94.7%)	8 (5.3%)	0.12–0.43 (mmol/L)

Blood parameter deltas on final screening					
Parameter	Remained normal	Normal to abnormal	Abnormal to normal	Remained abnormal	Reference value
WBC	133 (59.1%)	10 (4.4%)	70 (31.3%)	12 (5.3%)	4.00–10.00 (10^9^/L)
Hb	156 (69.3%)	9 (4.0%)	30 (13.3%)	30 (13.3%)	110–160 (g/L)
PLT	168 (74.7%)	6 (2.7%)	35 (15.6%)	16 (7.1%)	100–300 ()
ALT	129 (82.7%)	3 (1.9%)	6 (3.8%)	18 (11.5%)	0–40 (U/L)
AST	92 (59.0%)	14 (9.0%)	24 (15.4%)	26 (16.7%)	0–34 (U/L)
ALB	109 (69.9%)	14 (9.0%)	15 (9.6%)	18 (11.5%)	37–53 (g/L)
TBIL	95 (60.9%)	15 (9.6%)	17 (10.9%)	29 (18.6%)	5.1–19 (μmol/L)
DBIL	104 (66.7%)	15 (9.6%)	17 (10.9%)	20 (12.8%)	1.7–6.8 (μmol/L)
Urea	130 (83.9%)	11 (7.1%)	11 (7.1%)	3 (1.9%)	2.82–8.2 (mmol/L)
CR	121 (79.1%)	9 (5.9%)	11 (7.2%)	12 (7.8%)	45–104 (μmol/L)
UA	136 (89.5%)	5 (3.3%)	8 (5.3%)	3 (2.0%)	0.12–0.43 (mmol/L)

*Note: n* = 225 (WBC, Hb, Platelet), *n* = 156 (ALT, AST, ALB, TBIL, DBIL). *n* = 155 (UREA). *n* = 153 (CR). *n* = 152 (UA).

Abbreviations: ALB = albumin, ALT = alanine aminotransferase, AST = aspartate aminotransferase, CR = creatinine, DBIL = direct bilirubin, Hb = hemoglobin, PLT = platelet, TBIL = total bilirubin, UA = uric acid, WBC = white blood cell.

## 4 Discussion

In this study, we collected and analyzed the data of 8542 treatments of 251 patients with advanced tumors treated with Warburg therapy. The therapy was well tolerated with no serious adverse events requiring medical intervention nor were any post‐treatment hospitalizations necessary because of the therapy. During the hypoglycemia period induced with insulin, the vital signs of the patients were stable, and hypoglycemic symptoms were mild and completely reversed upon return to euglycemia. Most patients' weight was stable or improved throughout the course of their treatment as were blood indices of most patients with just over 90% of recorded patients' WBC and platelets in normal ranges post‐treatment.

Even though great strides in poverty alleviation in the past 40 years have been made with China accounting for 70% of world poverty reduction [18], cancer care costs continue to rise and present a real challenge for public health, especially in low‐income populations. A 2016 survey found that the average cost in the United States of a 6‐month standard‐of‐care chemotherapy regimen, many of which use off‐patent drugs, was approximately $27,000 USD [19] with newer in‐patent drugs costing approximately $24,000 a month [5]. Ten treatments for a course of treatment is the most over a typical one‐ to two‐month period, costs just $140 for supplies in addition to the cost of routine blood tests and a little ECG monitoring, and requires little to no supportive care due to the lack of serious adverse events. This therapy presents a real opportunity to deliver low‐cost to patient populations that otherwise face cost‐prohibitive access to care.

The basis for this low‐cost protocol is derived from the reliance of cancer cells on aerobic glycolysis over oxidative phosphorylation requiring large amounts of glucose to sustain their unlimited proliferation [20]. This altered metabolism of cancer being a hallmark of cancer has made it a major target for anticancer therapeutics although viable pharmacological agents to attack cancer's glycose metabolism have been elusive and remained mostly in the preclinical stage [9, 10, 11, 12, 13, 14, 15]. Some results have been studied using dietary approaches that restrict carbohydrate intake, such as ketogenic diets or fasting [21, 22, 23, 24], but dietary interventions cannot be scaled up because of the major compliance problems in patients due to their lengthy process, as well as malnutrition that compromises the immune system. The Warburg therapy's use of insulin pharmacologically induces hypoglycemia overcoming dietary compliance issues while creating a period where cancerous tumors are deprived of much of their needed glycose so they can be treated with chemotherapy. Studies have shown that tumor cells have more insulin receptors than normal cells, so using insulin to lower blood sugar can make chemotherapy drugs more effective. Cyclophosphamide is a broad‐spectrum antitumor drug, and studies have shown that low doses of cyclophosphamide can selectively eliminate regulatory T cells, resulting in immune activation. Compared with other chemotherapeutics, fluorouracil has a shorter half‐life and is more easily metabolized [25, 26]. Therefore, we choose the combination of these two chemotherapy agents and short‐term hypoglycemia to make it play a better effect and minimize the side effects. This innovative approach deprives tumor cells of energy and makes them better killed by chemotherapy drugs. The excellent safety profile allows the Warburg therapy to be used in conjunction with other therapies, such as immunotherapy, as this therapy seems to have little impact on WBC counts.

This article is the first of its kind in the international arena to report on the safety of a large number of treatments with the Warburg protocol. This report focuses on the clinical safety and affordability of the Warburg protocol. A common clinical observation with this therapy is a reduction in pain. This observation and the mechanisms of efficacy of Warburg therapy will be discussed in a subsequent article. We will systematically report the efficacy of this therapy and compare its effect with different hypoglycemic methods such as metformin.

## Author Contributions

**Peihua Lu:** conceptualization (equal), data curation (equal), resources (equal), supervision (equal), writing – original draft (equal), writing – review and editing (equal). **Tom Tsang:** formal analysis (equal), methodology (equal), resources (equal). **Michael S. Badowski:** writing – review and editing (equal). **Michael E. Pennington:** writing – review and editing (equal). **Linda C. Meade‐Tollin:** writing – review and editing (equal).

## Ethics Statement

All human studies were approved by the Ethics Review Committee of Xinglin Hospital of Traditional Chinese Medicine of Shenqiu City (SQ[2021‐6‐25]), and all participants obtained informed consent for the publication of human studies and related examinations.

## Conflicts of Interest

The authors declare no conflicts of interest.

## Translational Relevance

There are many options available for the treatment of advanced tumors. However, the benefits of these treatments for patients with advanced cancer are limited. Tumor growth is dependent on the Warburg effect; in the absence of glucose, tumor cells do not receive enough energy to support their growth and metastasis. Based on this, we designed Warburg therapy (i.e., using Warburg theory, glucose starvation was induced by intravenous insulin injection during cancer chemotherapy). It enhances the tumor cell killing effect through insulin‐induced hypoglycemia in combination with chemotherapy. An analysis of 8542 treatments in 251 patients with advanced cancer showed that Warburg therapy is clinically safe and inexpensive. This study provides evidence to support the safety of Warburg therapy as a potential complementary or standalone treatment option for patients with advanced tumors.

## Supporting information

Data S1.

## Data Availability

The authors confirm that most of the data supporting the findings of this study are available in the article [and/or its Supporting Information]. Other data, such as patient admission history information, can be requested from the corresponding author and will not be disclosed if privacy or ethical restrictions are involved.
