# Supplementary material for: Evaluation of the Clinical Safety of the Low‐Cost Warburg Therapy for the Treatment of Patients With Advanced Cancers
Source: Cancer Med. 2024 Dec 4;13(23):e70469. doi: 10.1002/cam4.70469 (PMC11615646; doi:10.1002/cam4.70469)
Supplement: Supplementary file 1 — Data S1. [file CAM4-13-e70469-s001.docx]

Supplemental Material


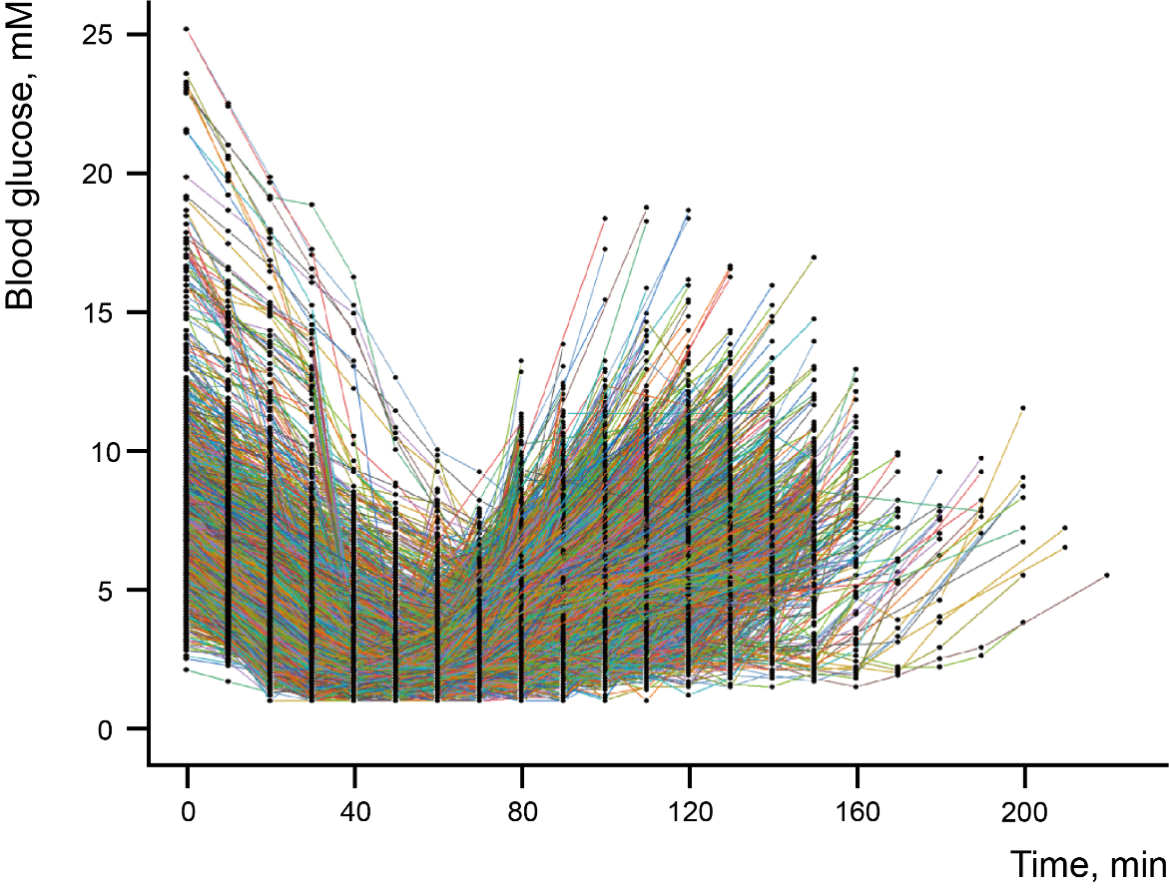


S1. Blood glucose fluctuates dynamically with time after a single treatment


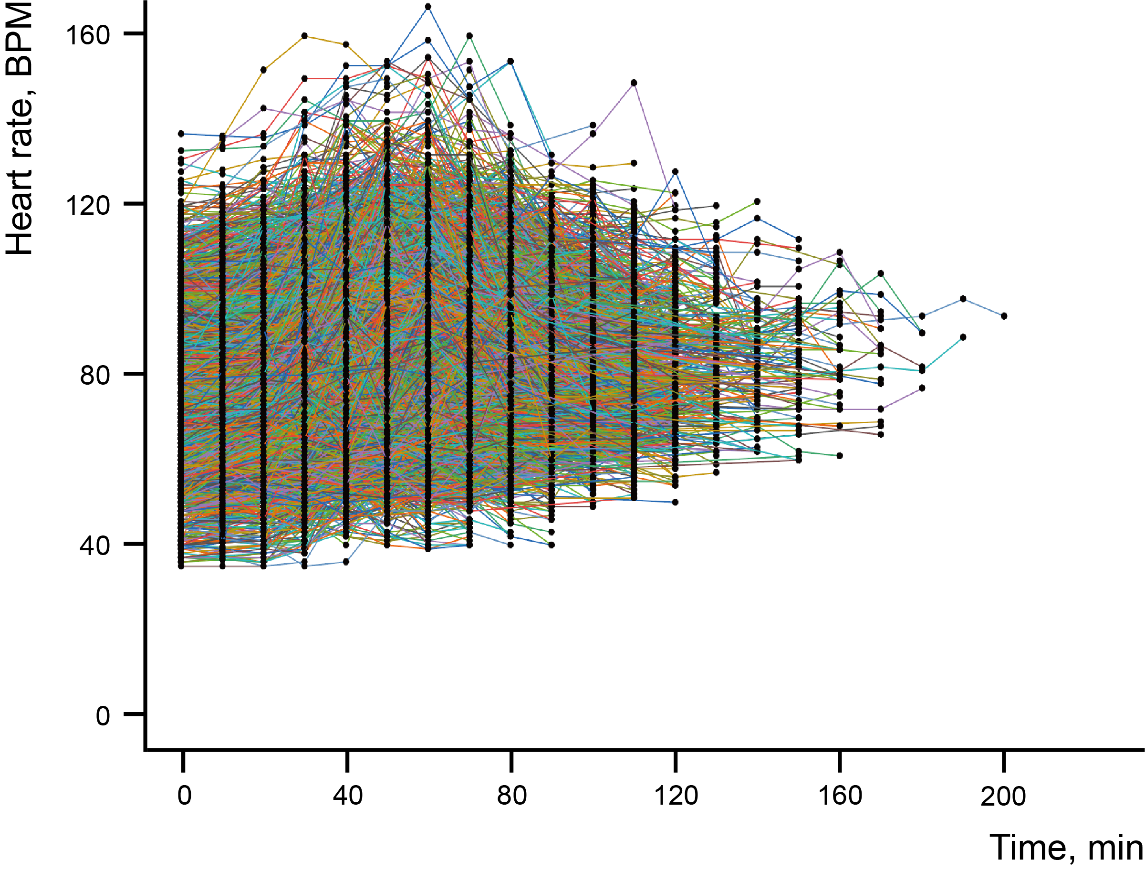


S2. The heart rate fluctuates dynamically with time after a single treatment


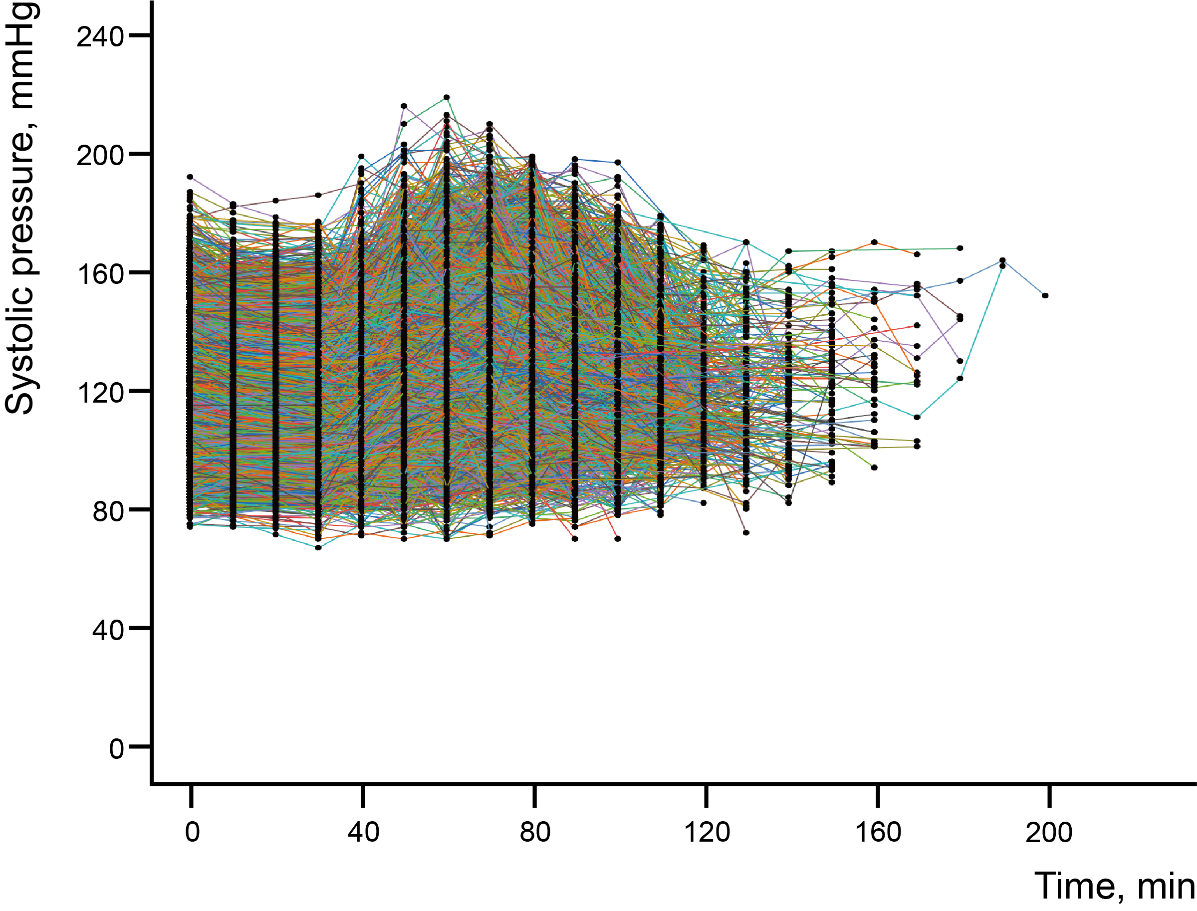
S3. Systolic blood pressure fluctuates dynamically with time after a single treatment


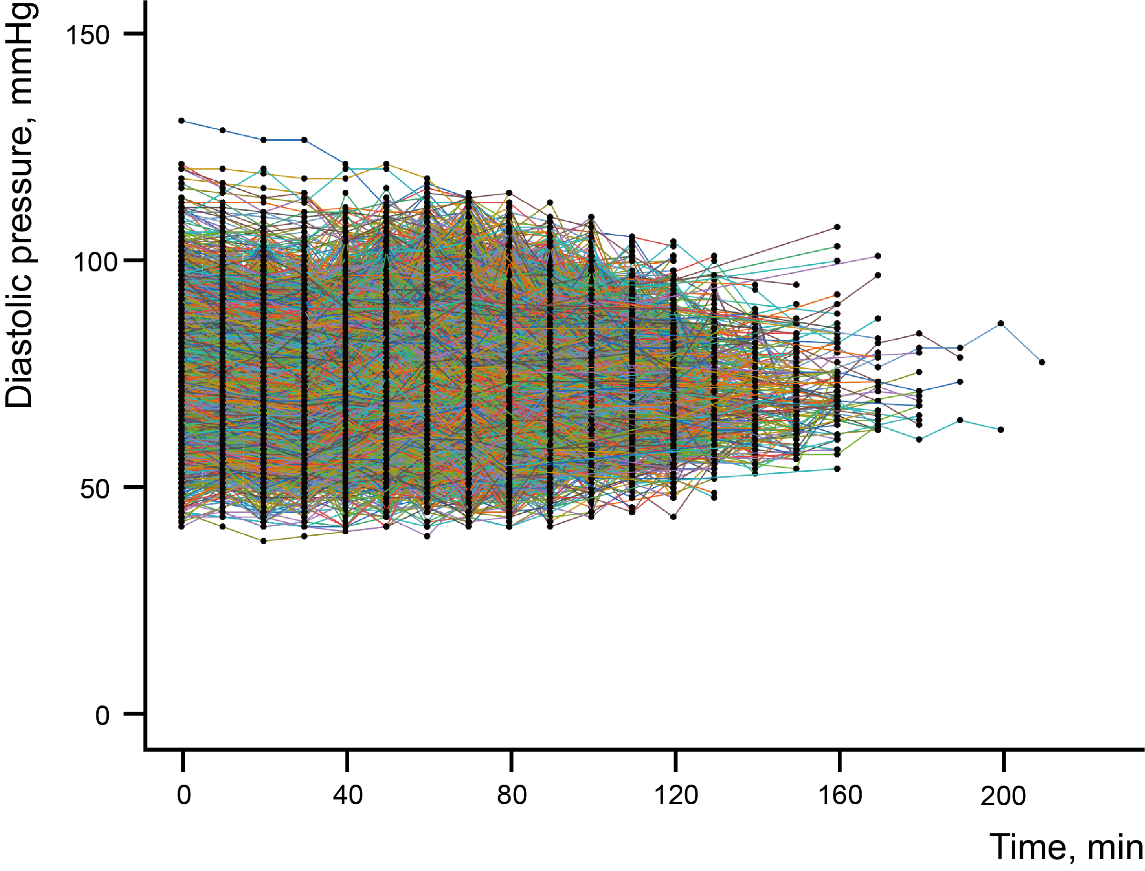
S4.Systolic blood pressure fluctuates dynamically with time after a single treatment


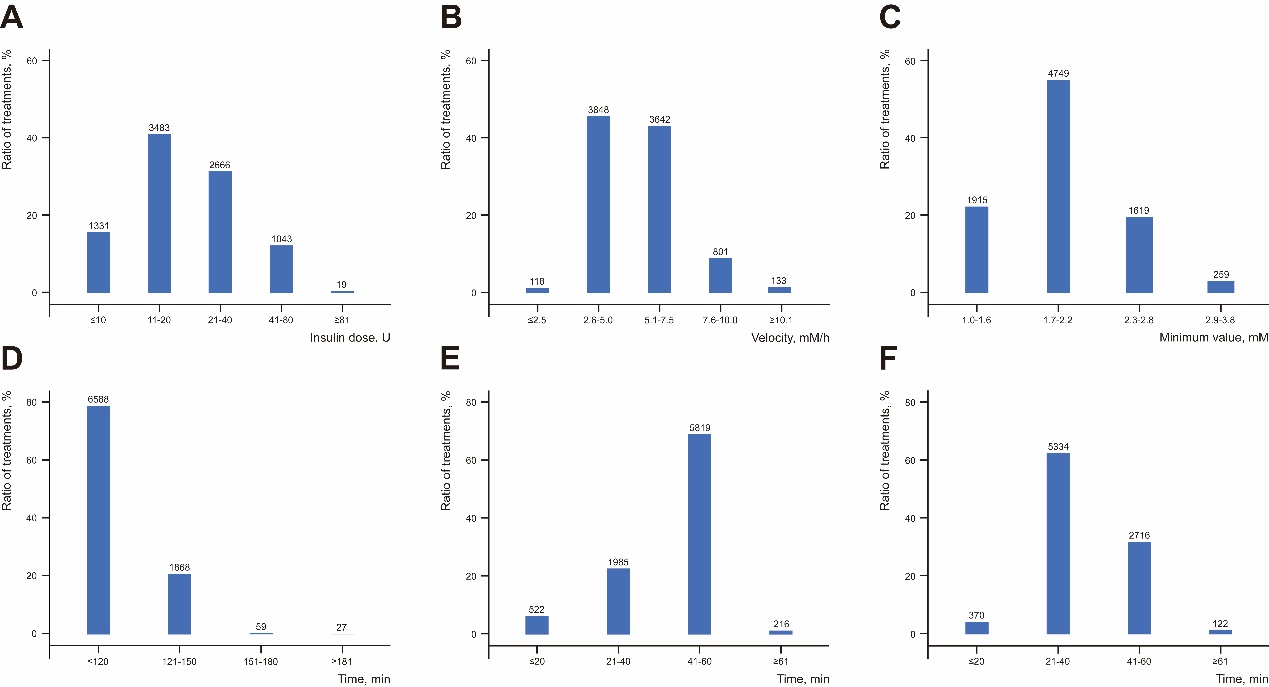


S5. single treatment insulin dosage and single treatment blood glucose lowering statistics. a: patient's single treatment insulin dosage statistics; b: patient's single treatment blood glucose lowering rate; c: patient's single treatment lowest blood glucose value; d: patient's single treatment total time; e: patient's single treatment hypoglycemia duration; f: patient's single treatment blood glucose recovery time


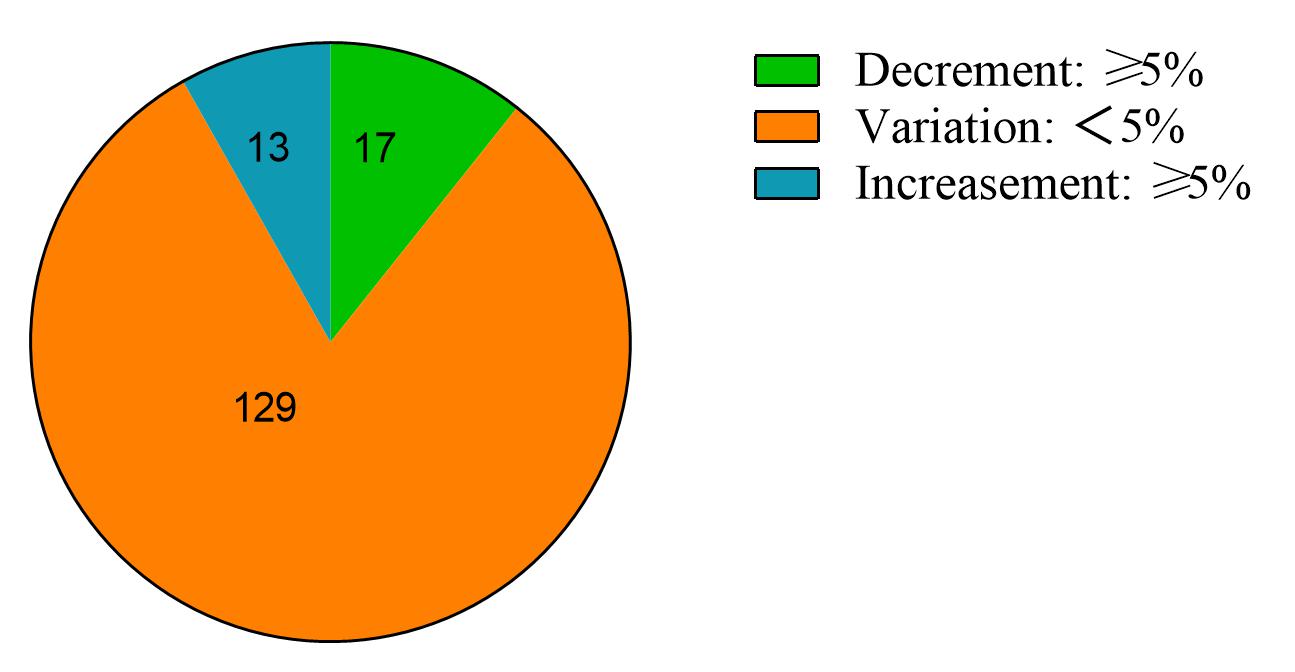


S6: Weight changes of patients before and after two courses of treatment
